# A SARS-CoV-2 Negative Antigen Rapid Diagnostic in RT-qPCR Positive Samples Correlates With a Low Likelihood of Infectious Viruses in the Nasopharynx

**DOI:** 10.3389/fmicb.2022.912138

**Published:** 2022-07-27

**Authors:** Isadora Alonso Corrêa, Débora Souza Faffe, Rafael Mello Galliez, Cássia Cristina Alves Gonçalves, Richard Araújo Maia, Gustavo Peixoto da Silva, Filipe Romero Rebello Moreira, Diana Mariani, Mariana Freire Campos, Isabela de Carvalho Leitão, Marcos Romário de Souza, Marcela Sabino Cunha, Érica Ramos dos Santos Nascimento, Liane de Jesus Ribeiro, Thais Felix Cordeiro da Cruz, Cintia Policarpo, Luis Gonzales, Mary A. Rodgers, Michael Berg, Roy Vijesurier, Gavin A. Cloherty, John Hackett, Orlando da Costa Ferreira, Terezinha Marta Pereira Pinto Castiñeiras, Amilcar Tanuri, Luciana Jesus da Costa

**Affiliations:** ^1^Laboratório de Genética e Imunologia das Infecções Virais, Departamento de Virologia, Instituto de Microbiologia Paulo de Góes, Universidade Federal do Rio de Janeiro, Rio de Janeiro, Brazil; ^2^Departamento de Doenças Infecciosas e Parasitárias, Instituto de Biofísica Carlos Chagas Filho, Universidade Federal do Rio de Janeiro, Rio de Janeiro, Brazil; ^3^Faculdade de Medicina, Universidade Federal do Rio de Janeiro, Rio de Janeiro, Brazil; ^4^Laboratório de Virologia Molecular, Instituto de Biologia, Universidade Federal do Rio de Janeiro, Rio de Janeiro, Brazil; ^5^Abbott Laboratories Inc., Chicago, IL, United States

**Keywords:** SARS-CoV-2, antigen rapid test, infectious virus detection, qRT- PCR, virus transmissibility

## Abstract

Severe acute respiratory syndrome-related coronavirus (SARS-CoV-2) transmission occurs even among fully vaccinated individuals; thus, prompt identification of infected patients is central to control viral circulation. Antigen rapid diagnostic tests (Ag-RDTs) are highly specific, but sensitivity is variable. Discordant RT-qPCR vs. Ag-RDT results are reported, raising the question of whether negative Ag-RDT in positive RT-qPCR samples could imply the absence of infectious viruses. To study the relationship between negative Ag-RDT results with virological, molecular, and serological parameters, we selected a cross-sectional and a follow-up dataset and analyzed virus culture, subgenomic RNA quantification, and sequencing to determine infectious viruses and mutations. We demonstrated that RT-qPCR positive while SARS-CoV-2 Ag-RDT negative discordant results correlate with the absence of infectious virus in nasopharyngeal samples. A decrease in sgRNA detection together with an expected increase in detectable anti-S and anti-N IgGs was also verified in these samples. The data clearly demonstrate that a negative Ag-RDT sample is less likely to harbor infectious SARS-CoV-2 and, consequently, has a lower transmissible potential.

## Introduction

Since December 2019, more than 250 million confirmed cases and 5 million deaths have been attributed to severe acute respiratory syndrome-related coronavirus (SARS-CoV-2)-related infections worldwide (World Health Organization, 2020). Vaccines against COVID-19 became available at the end of 2020, and as of 12 November 2021, 3.1 billion people were fully vaccinated (41% of the world's population). Although vaccination may reduce COVID-19 cases and disease severity (Hall et al., 2021; Shah et al., 2021), SARS-CoV-2 breakthrough infection still occurs among fully vaccinated subjects (Bailly et al., 2021; Bergwerk et al., 2021; Chau et al., 2021). In this scenario, social distancing measures and early detection/isolation of infected individuals remain central for viral dispersion control. SARS-CoV-2 diagnosis relies on the gold-standard technique of nucleic acid amplification tests (NAATs), and several assays targeting different viral genes are available and widely used (CDC, 2020). In Brazil, the Health Department recommends the use of NAATs within 3–15 days from the beginning of symptoms only for hospitalized patients with severe respiratory syndrome, healthcare workers presenting flu-like symptoms, and organ donation candidates (Larremore et al., 2021). However, the high costs and operational requirements limit its availability. Besides, NAATs long turnaround time to get results may negatively impact the clinical outcome and epidemiology. Thus, strategies to accelerate diagnosis are still required.

SARS-CoV-2 Ag-RDTs represent powerful tools to expand the number of people tested and increase testing frequency (Mina et al., 2020; Larremore et al., 2021; Pilarowski et al., 2021). Most commercial Ag-RDTs, based on lateral flow immunoassays, capture SARS-CoV-2 nucleocapsid (N) protein due to their high degree of conservation and the proportionally high amounts in coronavirus virions (Wang et al., 2020; Diao et al., 2021).

Currently, WHO recommends the use of Ag-RDTs as a diagnostic tool when the tests achieve a minimum of 80% sensitivity and 97% specificity, compared with a reference NAAT. Ag-RDTs can be especially useful in locations where NAAT is unavailable or has delayed time responses (World Health Organization, 2021). In fact, several studies report high specificity with few variations among Ag-RDT assays in clinical samples (Linares et al., 2020; Corman et al., 2021; Igloi et al., 2021; Pilarowski et al., 2021; Routsias et al., 2021). Most studies also demonstrate a correlation between the number of days since symptom onset (DSSO), viral load, and sensitivity. Ag-RDTs' sensitivity varies from 85 to 94% when used 3–5 DSSO (Chaimayo et al., 2020; Linares et al., 2020; Igloi et al., 2021) with high sensitivity for samples with *C*_t_ values below 25 (Chaimayo et al., 2020; Igloi et al., 2021; Korenkov et al., 2021).

In addition to its low cost and quick result, preliminary studies have also demonstrated a reliable correlation between a positive Ag-RDT result and viral isolation in cell culture, supporting Ag-RDTs as a convenient tool for early detection of the high virus spreading individuals, enabling appropriate patient isolation, and improving transmission control (Pekosz et al., 2021; Pickering et al., 2021). Although its cause remains unclear, a small proportion of patients show a negative Ag-RDT result with detectable viral load by NAAT and positive viral culture (Homza et al., 2021). Thus, these discordant cases (Ag-RDT-/NAAT+) could impact the spread of SARS-CoV-2 (Homza et al., 2021; Pray et al., 2021).

In this study, we analyzed patients with positive SARS-CoV-2 RT-qPCR results and negative Ag-RDT using the Panbio™ COVID-19 Ag test (Abbott) from a cohort of patients attending for SARS-CoV-2 diagnosis at the Federal University of Rio de Janeiro, Brazil, from August 2020 to September 2021. We found that 10–35% of samples that tested positive for SARS-CoV-2 RT-qPCR were negative at Ag-RDT consistently throughout the period of the study, regardless of the predominant SARS-CoV-2 variant circulating in the Rio de Janeiro State. We further characterized 23 concordant (RT-PCR+/Ag-RDT+) and 29 discordant (RT-PCR+/Ag-RDT-) samples for virus isolation in cell cultures, RT-qPCR correlation, presence of anti-Spike and anti-nucleocapsid IgG, full-length viral genome, viral sub-genomic mRNA (sgRNA), and viral protein content. We found that discordant samples harbor intact full-length genomes, but only 10.53% were replication-competent compared with 56.52% of the true positive counterpart. Through statistical models and *in vitro* assays, we demonstrated that the two variables with greater predictable capacity for a positive Ag-RDT result were the RT-qPCR *C*_t_ value and the absence of humoral response against SARS-CoV-2.

## Materials and Methods

### Study Samples

The Federal University of Rio de Janeiro offers diagnostic tests for mildly symptomatic public healthcare and security force workers in the city of Rio de Janeiro and to the University community. For patients presenting up to 5 days from symptom onset at the time of testing, a nasopharyngeal swab was taken from one patient's nostril to perform the Panbio™ COVID-19 Ag test using the swab provided with the kit. Additional nasopharyngeal swabs were collected from both nostrils using two rayon-tipped swabs for RT-qPCR testing. The viral transport medium (VTM) sample was then used for RNA extraction and RT-qPCR for SARS-CoV-2 detection, and the leftover sample was stored at −80°C for further inoculation in cell culture. All patients were requested to take another RT-qPCR test 14 days after the initial symptoms and, if it remained positive, to take a follow-up test every 7 days until a negative RT-qPCR result was obtained. Plasma and serum were also collected during each visit for serological test purposes.

**Figure 1:**
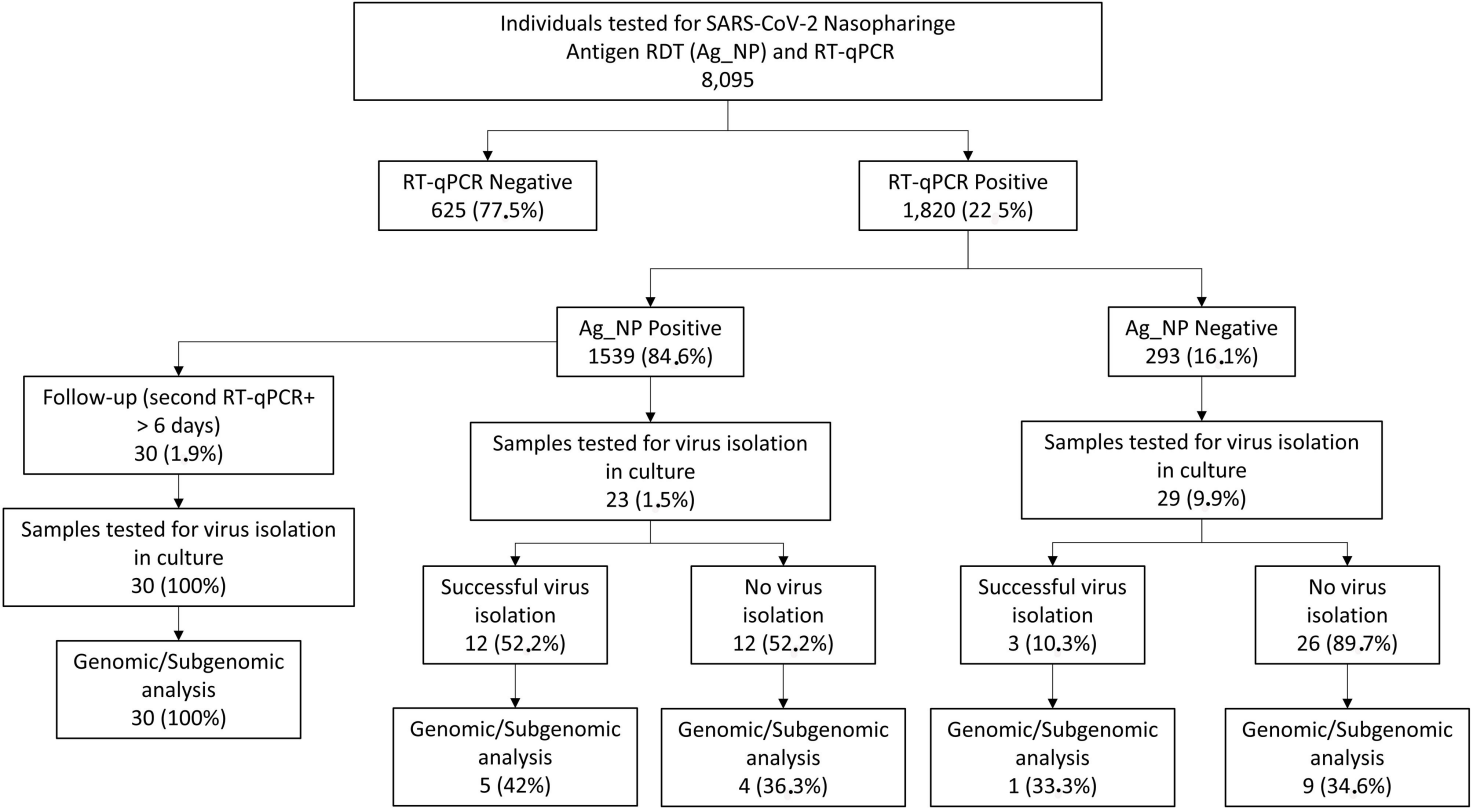
Schematic representation of the UFRJ-CTD cohort from 20 August to 21 September and sample selection. Mildly symptomatic individuals tested for severe acute respiratory syndrome-related coronavirus (SARS-CoV-2) diagnosis at the Center for COVID-19 Diagnosis of Federal University of Rio de Janeiro from August 2020 to September 2021 account for a total of 8.095 RT-qPCR and antigen RTD exams. Of this total, 1.820 tested positive for SARS-CoV-2 1.539 also being antigen RDT positive (concordant samples) and 293 antigen RDT negative (discordant samples). From this group, 61 samples (23 concordant and 29 discordant) were selected for further studies comprising the cross-sectional dataset while 30 additional patients were selected for the follow-up dataset where the patient has a second sample collected with a minimum of 6 days after diagnosis.

We selected samples from August 2020 to September 2021 in two study cohorts: a cross-sectional cohort composed of patients that, at the time of diagnosis, presented an antigen test that was further confirmed (antigen concordant) or not (antigen discordant) by RT-qPCR and a follow-up cohort, composed of patients that, at the time of diagnosis, presented antigen, and RT-qPCR positive results and had at least one more sample collected with a minimum 6-day interval where antigen and RT-qPCR results were concordant or discordant. For all the selected samples, 100 μl of the VTM sample was tested again with the Panbio™ COVID-19 Ag test according to the manufacturer's instructions. A flow chart of the sample selection and the study's cohorts is shown in Figure 1.

This study was approved by the National Committee of Research Ethics (CAAE-30161620.0.1001.5257). All enrolled participants were above 18 years old and declared written informed consent.

### Cell Lines

African green monkey kidney cells (Vero E6, ATCC CRL-1586) and the humanized Vero expressing hAce-2 and hTMPRSS2 (ATCC), referred to as Vero-hA/T, were maintained in DMEM (ThermoFisher Scientific) supplemented with 10% fetal bovine serum (FBS; Gibco), 100 U/ml penicillin, and 100 μg/ml of streptomycin (ThermoFisher Scientific). The cells were incubated at 37°C with 5% of CO_2_ and passed every 3–4 days.

### Viral Isolation

For viral isolation, Vero E6 or Vero-hA/T was plated in six well plates 24 h before inoculation to achieve 70% confluence overnight. Cells were infected with 250 μl of VTM diluted in DMEM plus 300 U/ml penicillin and 300 μg/ml of streptomycin, with no addition of FBS for 1 h for viral adsorption. After the adsorption period, 10% FBS-supplemented DMEM with 300 U/ml penicillin and 300 μg/ml streptomycin was added to the inoculum, and the cells were incubated at 37°C and 5% CO_2_ for 72 h (passage #1). The supernatants of passage #1 were collected, 250 μl of supernatants was used to infect fresh cell cultures as previously described and incubated for another 72 h (passage #2), and the remaining supernatant was stored at −80°C for RNA extraction and RT-qPCR for SARS-CoV-2 detection. Passage #2 supernatants were also collected and stored at −80°C for viral titration, RNA extraction, and RT-qPCR for SARS-CoV-2 detection. For both cell passages, 100 μl of the collected supernatant was used to perform the Panbio™ COVID-19 Ag assay. For viral titration, 10-fold dilutions of each sample from passage #2 were used to infect Vero E6 cells. After 1 h of virus adsorption, media was replaced by fresh DMEM with 1% FBS, 100 μ/ml penicillin, 100 μg/ml streptomycin, and 1.4% carboxymethylcellulose (Sigma Aldrich) followed by incubation for 4 days at 37°C with 5% CO_2_. Then, cells were fixed with 10% formaldehyde and stained with 1% crystal violet in 20% methanol for plaque visualization and quantification. Viral titers were expressed as plaque-forming units per milliliter (PFU/ml). Positive viral isolation was defined by the visualization of viral plaques after titration of the passage #2 supernatants. If a sample presented a drop of *C*_t_ value from cell passage #1 to cell passage #2, we performed an additional cell passage (passage #3) as previously described. We considered the sample positive for virus isolation once plaques were visualized in the titration assay. All the experiments were conducted in a BSL-3 laboratory.

### RNA Extraction

RNA was extracted from 200 μl of cell culture supernatant from cell passages #1, #2, and #3 when available, using the ReliaPrep™ Viral TNA Miniprep System (Promega) according to the manufacturer's instructions. RNA was eluted in 50 μl and stored at −80°C until RT-qPCR.

### RT-qPCR

For detection of genomic and subgenomic SARS-CoV-2 RNA, we used viral RNA extracted from VTM at the time of diagnosis. Genomic RNA was detected using SARS-CoV-2 (2019-nCoV) CDC qPCR Probe Assay (Integrated DNA Technologies, IA, USA) targeting the SARS-CoV-2 N gene and Brilliant III Ultra-Fast qRT-PCR Master Mix (Agilent). For subgenomic RNA, specific primers for gene N subgenomic product were used together with a probe for N gene along with Brilliant III Ultra-Fast qRT-PCR Master Mix (Agilent) as follows: 0.08 μl of primer forward; 0.08 μl of reverse primer; 0.04 μl of probe; 10 μl of 2 × master mix; 0.2 μl of 100 mM DTT; 0.3 μl of reference dye (dilute 1:500); 1.0 μl of RT/RNase block; 3.0 μl of nuclease-free water, and 5 μl of RNA. The cycling used for subgenomic detection was 50°C for 10 min; 95°C for 3 min; 45 cycles of 95°C for 15 s, and 53°C for 30 s. Sequences of primers and probes are described in Supplementary Figure 1. For the detection of genomic RNA from viral cell cultures, we used the AnGene Kit according to the manufacturer's instructions. All the reactions were performed using the AriaMX real-time PCR System (Agilent).

### Sequencing and Phylogenetic Analysis

Illumina sequencing proceeded using two distinct protocols for library construction, as described in Orf et al. (2021) and Moreira et al. (2021). In brief, viral RNA was converted to cDNA with SuperScript IV (ThermoFisher, USA), and whole-genome amplification was performed with the ARTIC SARS-CoV-2 V3 primer panel and the Q5 hot-start polymerase (NewEnglandBiolabs, USA). Amplicons were purified and converted into Illumina sequencing libraries with the QIAseq FX library kit (QIAGEN, Germany), following the manufacturer's protocol. Libraries were quantified with the Qubit dsDNA HS kit and equimolarly pooled, being sequenced on an Illumina MiSeq run with a V3 (600 cycles) cartridge (Illumina, USA).

Raw sequencing data were processed, and reference assemblies were performed using a custom pipeline. Whole-genome sequences were then classified using both the pangolin tool version 3.1.14 (Pango version 1.2.81) (O'Toole et al., 2021) and NextClade version 1.7.3 (Hadfield et al., 2018). Later, to further contextualize the novel genome sequences, a maximum likelihood phylogenetic inference was performed with IQ-Tree version 2.0.3 (Minh et al., 2020), under the GTR+F+I+G4 model. The reference dataset was assembled by querying the GISAID EpiCoV database to retrieve the 50 sequences most similar to each of ours, using the Audacity-Instant application (Acknowledgement table available in Supplementary File 1). After removing duplicates, the final dataset comprehended 555 genome sequences. All sequences generated in this study were deposited in GISAID. The GISAID accession codes (EPI_ISL_11836071 through EPI_ISL_11836089; and EPI_ISL_4413334 - EPI_ISL_4413337) together with other sequence information are listed in Supplementary Table 1.

### Western Blotting

For protein analyses, infected-whole cell lysates were collected with RIPA buffer (10 mM Tris-Cl [pH 8.0]; 1 mM EDTA; 0.5 mM EGTA; 1% Triton X-100; 0.1% sodium deoxycholate; 0.1% SDS; and 140 mM NaCl) mixed with 0.1% protease inhibitor cocktail (Sigma-Aldrich). Proteins were fractionated by a 4–20% precast gel SDS-PAGE and blotted onto a nitrocellulose membrane (Hybond-ECL, GE Healthcare), using a wet tank transfer system (BioRad). Membranes were probed with primary antibodies, namely, anti-nucleocapsid (cat no: 26369, Cell Signaling) and anti-spike (cat no: 56996, Cell Signaling). The secondary antibodies used were the horseradish peroxidase HRP-conjugated Anti-Rabbit (KPL). SuperSignal West Pico Chemiluminescent Substrate (Thermo Scientific) was used as a substrate for the horseradish peroxidase (HRP) chemiluminescent reaction.

### Serological Assay

Microtitre plates (Immulon 2 HB) were coated with a trimeric mammalian-culture secreted ectodomain of the Spike protein—Wuhan in the pre-fusion conformation (Alvim et al., 2021) at 4 μg/ml or the nucleocapsid—Wuhan protein produced as described by dos-Santos et al. (2021) at 1 μg/ml in 100 mM sodium carbonate-bicarbonate buffer (pH 9.6) and incubated overnight at 4°C. Excess protein was removed by washing five times with PBS + 0.05% Tween-20 (PBST, Sigma), and unbound sites were blocked using 5% BSA in PBST. Samples were added at 1/50 dilution in PBST + 2% BSA, followed by 1 h at 37°C. The plates were washed five times with PBST, and a polyclonal anti-human IgG antibody conjugated to HRP (Promega) was added for 1 h at room temperature. Plates were washed five times with PBST followed by the addition of the chromogenic substrate TMB (Sigma) for 10 min and the reaction stopped with 1N sulfuric acid. Absorbance was read at 450 nm with an ELISA microplate reader (Biochrom Asys). A positive control specimen (from a known COVID-19 patient) in simplicate and a negative control (pre-epidemic plasma sample) in triplicate were added to every assay plate for validation and cutoff determination. Results were expressed as a reaction of the sample optical density value divided by assay cutoff.

### Statistical Analysis

The data from the cohort were acquired using the KoBoCollect online/offline web-based form (available at: https://www.kobotoolbox.org). The data were extracted as a dataset based on XLSForms and merged with the laboratory data. Differences between concordant and discordant groups were assessed by Mann–Whitney nonparametric *U*-test. A multivariate model based on logistic regression was applied for multivariate analysis. GraphPad Prism version 9.2.0 (GraphPad Software, San Diego, California USA), JASP version 0.16 (JASP Team, 2021) [Computer software]), and R (R Core Team 2021 *R: A Language and Environment for Statistical Computing*, Vienna, Austria available at: https://www.R-project.org/) were used. A *p*-value <0.05 was considered significant.

## Results

From August 2020 to September 2021, a total of 8,095 individuals were simultaneously tested for SARS-CoV-2 by RT-qPCR and Panbio™ Antigen RDT tests (Figure 1) at the center for COVID-19 diagnosis at the Federal University of Rio de Janeiro. While 1,820 were positive for RT-qPCR (22.5%), 1,539 (20.34%) were positive for the Ag-RDT (Figure 1), yielding 84.6% of concordant (RT-qPCR+/Ag-RDT+) and 16.1% of discordant (RT-qPCR+/Ag-RDT-) samples (Figure 1). The average age was 40.4 and 38.4 years, with 58 and 54% of women, in the concordant and discordant groups, respectively. Odds ratio measurement demonstrated no association of each demographic variable with a concordant (RT-qPCR+/Ag-RDT+) or discordant (RT-qPCR+/Ag-RDT–) status (Supplementary Table 2). All individuals presented mild symptoms in both groups, and the most frequent comorbidity was hypertension (17.9 vs. 15.5% in concordant and discordant groups, respectively). Immunodeficiency had a low frequency among these individuals with 0.4 vs. 1.1% in concordant and discordant groups, respectively. No association of specific symptoms or comorbidity with a concordant (RT-qPCR+/Ag-RDT+) or discordant (RT-qPCR+/Ag-RDT-) status was observed (Supplementary Table 2).

A multivariate logistic regression model applied to variables such as gender, age, and symptoms confirmed that none were associated with either the concordant or the discordant groups (Supplementary Figure 2 and Supplementary Table 3).

**Figure 2:**
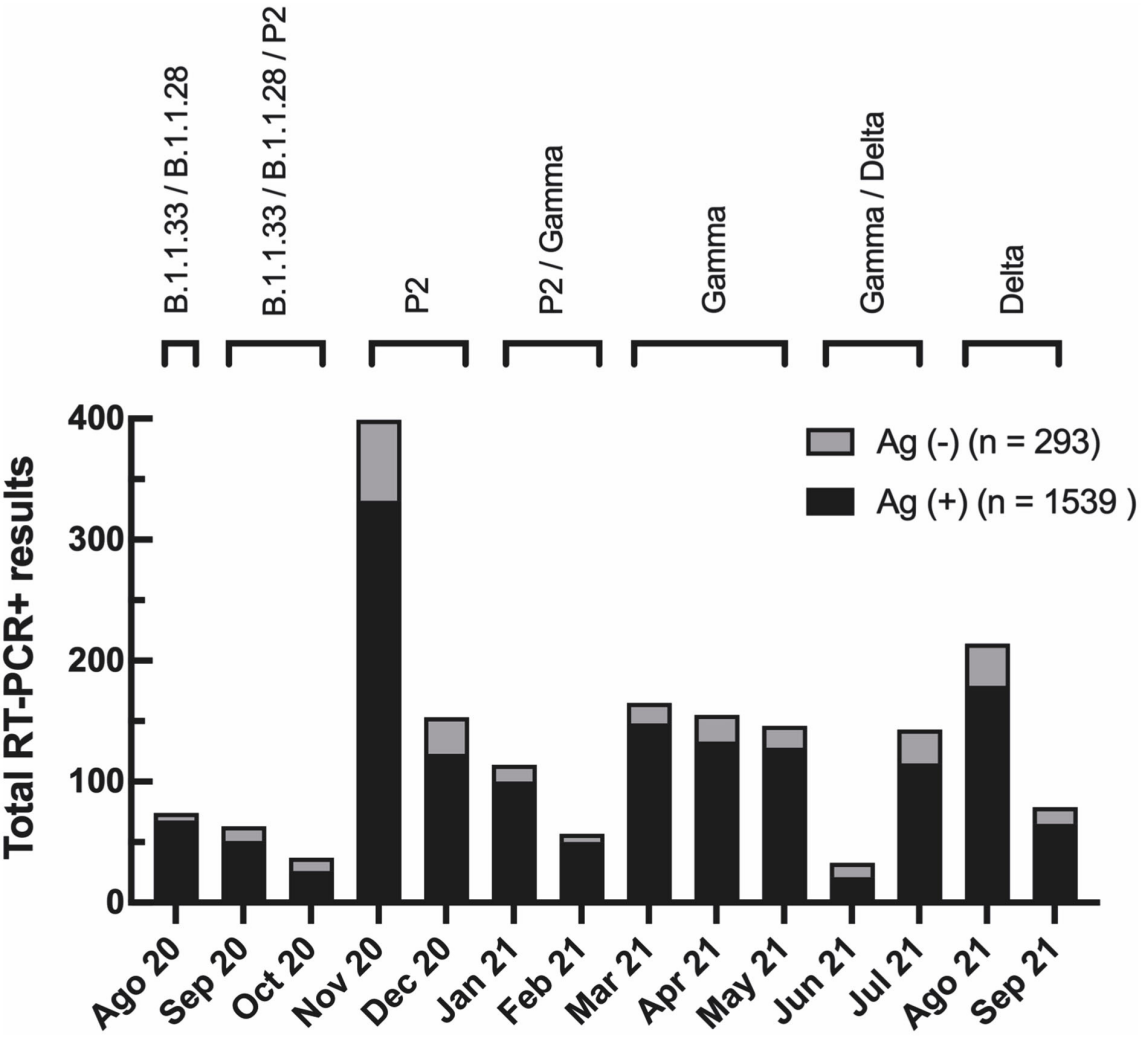
Evaluation of Panbio™ COVID-19 Antigen RDT test showed low false-negative occurrence with no variation during the time of the study. The total number of RT-PCR positive results per month in the studied population. In black, the total number of RT-PCR positive individuals with a negative antigen [Ag (–)] RDT result (discordant group). In gray, the total number of RT-PCR positive individuals with a positive Antigen [Ag (+)] RDT result (concordant group). At the top, prevalent viral variants in Rio de Janeiro state during the study, according to data from the Vigilance Network of the State of Rio de Janeiro (blob:http://www.corona-omica.rj.lncc.br/4efa46e4-9323-452e-b3b2-c8014526a9ad).

Importantly, 72.9% of the individuals in the concordant group and 72.3% in the discordant group have not been vaccinated. The individual demographic data for the total number of participants diagnosed from August 2020 to September 2021 in our cohort are shown in Supplementary Table 4. The total number of samples tested per month varied from 159 (June 2021) to 1,208 (November 2020), with a positivity rate/month of 15% (September 2021) to 36% (November 2021). Regardless of the transmission rates, the percentage of discordant samples did not vary substantially over time (Figure 2). Different SARS-CoV-2 variants circulated in Rio de Janeiro during this period (Figure 2), suggesting that Ag-RDT sensitivity was not impacted by these viral variants and neither with by the beginning of the vaccination program in January 2021.

Using a logistic regression analysis model, we demonstrated that RT-qPCR *C*_t_ value, DSSO, and IgG anti-S significantly contribute to the probability of detecting a positive Ag-RDT. The RT-qPCR *C*_t_ value had the highest impact (Supplementary Figures 3, 4). The month of diagnosis could reflect a potential difference in the level of viral circulation, the prevalence of different variants over time, and the percentage of vaccinated individuals. However, it did not affect the probability of having a positive Ag-RDT result, suggesting that these factors did not alter Ag-RDT sensitivity (Supplementary Figure 4).

**Figure 3:**
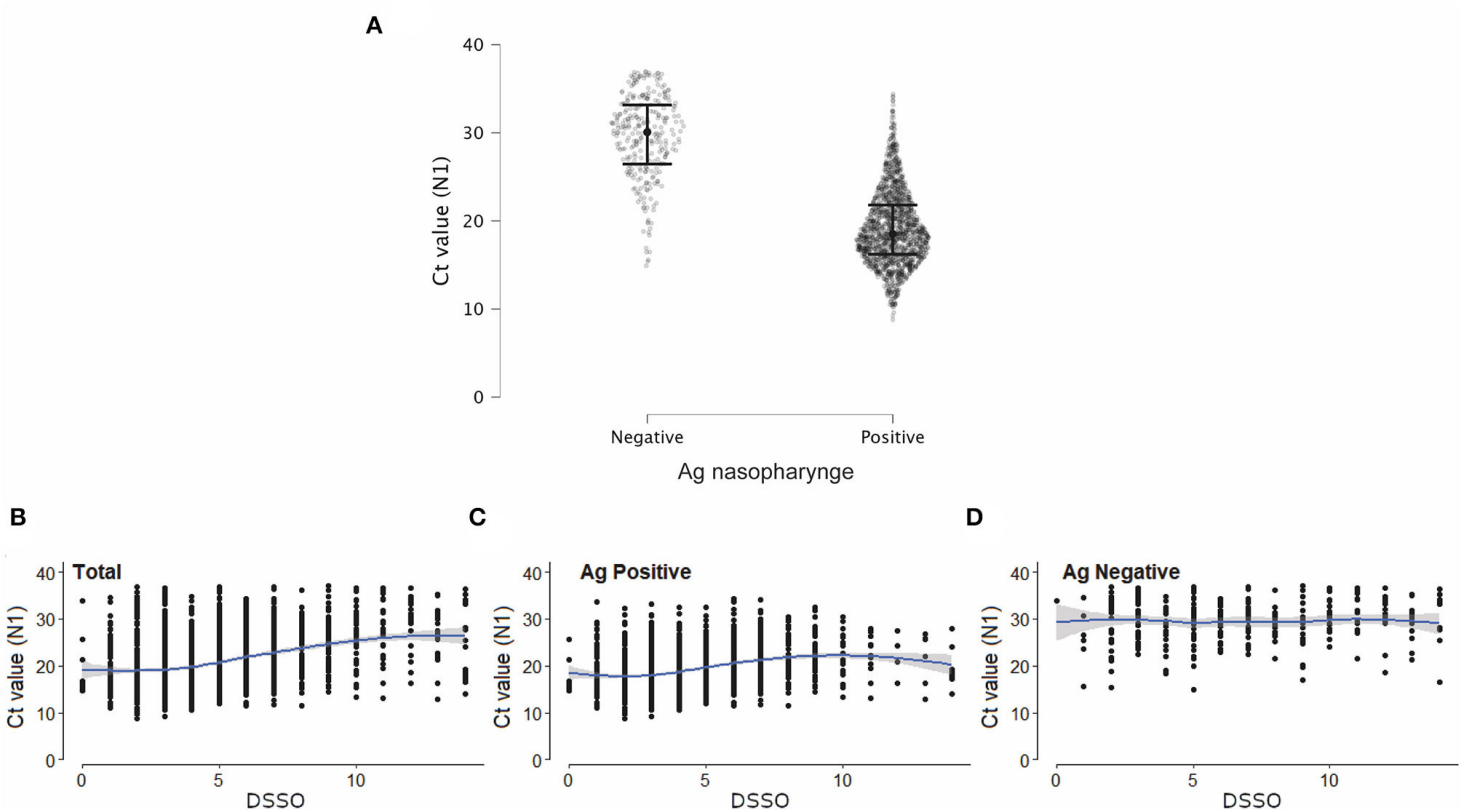
Discordant samples (RT-PCR+/Ag-RDT-) showed higher *C*_t_ values than concordant ones (RT-PCR+/Ag-RDT+). **(A)** *C*_t_ values (N1 target) of SARS-CoV-2 RT-PCR in patients with a positive (*n* = 1,539) or negative (*n* = 293) nasopharyngeal antigen (Ag) rapid diagnostic test (RDT) result tested from August 2020 to September 2021. **(B–D)** Correlation between *C*_t_ value (N1 target) of SARS-CoV-2 RT-PCR and days since symptom onset (DSSO) when samples were collected in total patients **(B)**, and among patients with a positive or negative Ag-RDT result (**C,D**, respectively).

Ag-RDT negative samples had higher *C*_t_ values (Figure 3A) than Ag-RDT positive ones. A positive correlation between *C*_t_ values and DSSO for all samples was observed (Figure 3B). This correlation was maintained for Ag-RDT positive (concordant) (Figure 3C) but not for Ag-RDT negative (discordant) samples (Figure 3D), probably due to their higher *C*_t_ values.

To further understand what influenced the sensitivity of the Ag-RDT when compared with the RT-qPCR assay, a total of 68 samples were selected (23 concordant and 29 discordant). The average age and the presence of mild disease symptoms were equivalents between the concordant and discordant groups (Supplementary Table 5). No participant had a previous diagnosis of immunodeficiency.

**Figure 4:**
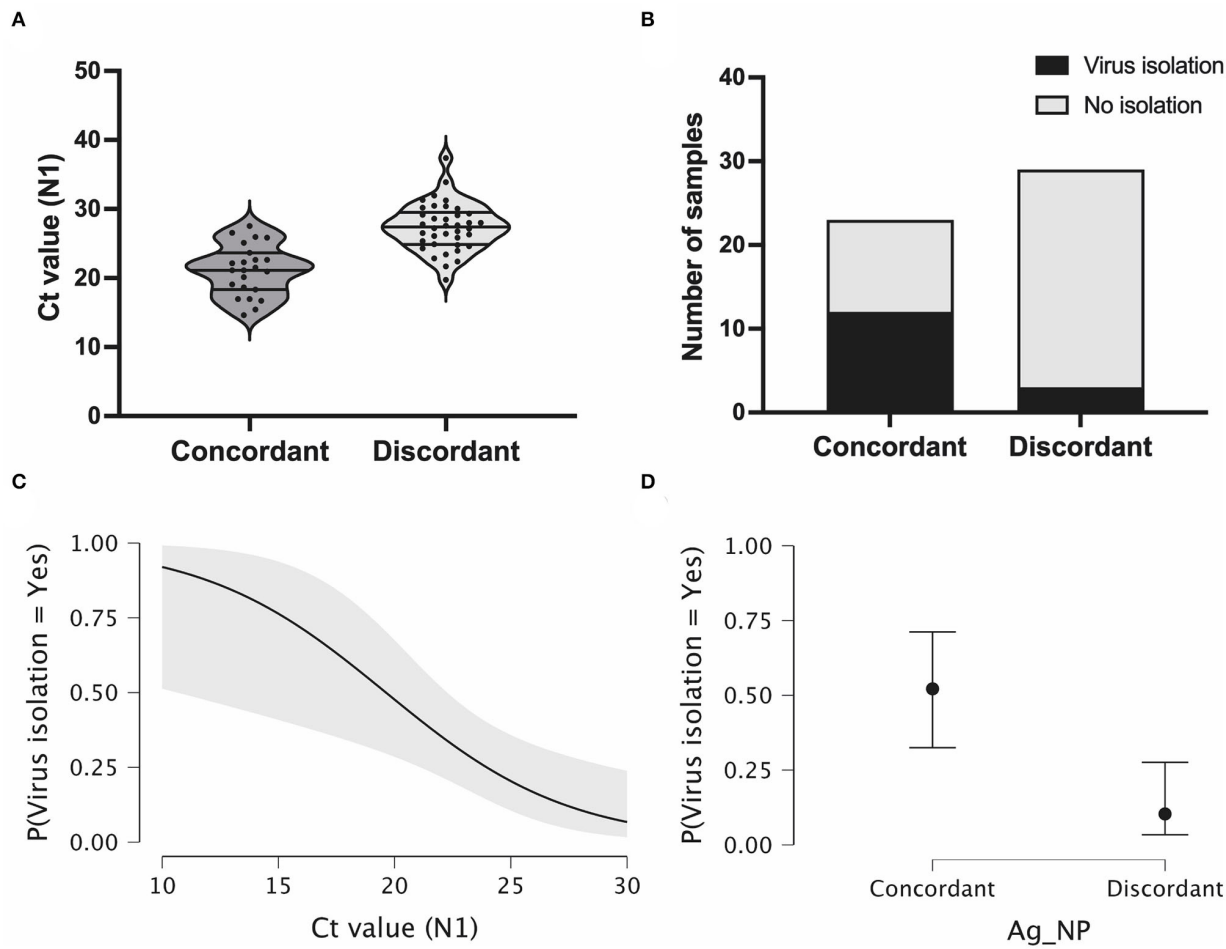
Antigen discordant samples have higher *C*_t_ and lower viral isolation rates compared with discordant samples. **(A)** Violin plot showing RT-PCR *C*_t_ values (N1 target gene) in antigen concordant (RT-PCR+/Ag-RDT+, *n* = 23) and discordant (RT-PCR+/Ag-RDT-, *n* = 29) samples used for virus isolation experiments, and lines represent the median and 25–75% quartiles. **(B)** Virus isolation success in total number of antigen concordant (*n* = 23) and discordant (*n* = 29) samples. **(C,D)** Viral isolation probability considering RT-PCR *C*_t_ values (N1 target gene) and nasopharyngeal antigen rapid test (Ag-NP) result as isolated covariates, respectively.

We analyzed the *C*_t_ value at the time of diagnosis for all samples in both study groups. Discordant samples notably had higher *C*_t_ values (20–38, median = 28) when compared with concordant samples (14–28, median = 21) (Figure 4A), as demonstrated above for the total number of samples (Figure 3A).

We evaluated the presence of viable infectious viruses in concordant and discordant samples after two consecutive cell passages. Positive virus isolation was only confirmed after virus titration using plaque assay. From the 23 concordant samples, 12 samples were positive for virus isolation (rate = 52.17%). In contrast, for the 29 discordant samples, only 3 were positive for virus isolation (rate = 10.34%) (Figure 4B). These percentages indicate a significant difference in the isolation success between Ag-/RT-PCR+ and Ag+/RT-PCR+ groups (*p* < 0.00001, CI 0.05). When the sample *C*_t_ is considered as an indicator of virus isolation success, the higher probability of virus isolation significantly occurred for both concordant and discordant samples in the low *C*_t_ range (Figure 4C) (Chaimayo et al., 2020; Linares et al., 2020; Wang et al., 2020; Corman et al., 2021; Diao et al., 2021; Igloi et al., 2021; Korenkov et al., 2021; Pickering et al., 2021; Pilarowski et al., 2021; Routsias et al., 2021; World Health Organization, 2021). This probability dropped to <25% with *C*_t_ values >26 (Figure 4D). When analyzing discordant and concordant samples separately for virus isolation success in the same *C*_t_ range, the probability of virus isolation was below 10% for the former and above 50% for the latter (*p* = 0.002).

The absence of viral particles in nasopharynges still positive for total viral RNA could be due, in major part, to the presence of viral subgenomic RNA (sgRNA). To verify the influence of sgRNA on the *C*_t_ value, we performed parallel target-specific RT-qPCR for genomic and N-sgRNA in a few concordant and discordant samples (Supplementary Figure 1A). We observed that discordant samples presented higher *C*_t_ values for both genomic and N-sgRNA than concordant samples (Supplementary Figure 1B). Since sgRNA is a marker of viral replication, these data suggest a lower level of replication in antigen discordant samples, which correlates with a lower proportion of these samples harboring infectious viruses. Importantly, from these data, we showed that N-sgRNA proportionally contributed 20% of the total amount of the viral RNA present in both concordant and discordant nasopharyngeal samples (Supplementary Figure 1C). These results suggest that RT-qPCR positive, but antigen-negative nasopharyngeal samples, lack competent/infectious viral particles in part due to a lower level of viral replication, thus having a lower impact on the spread of SARS-CoV-2.

**Figure 5:**
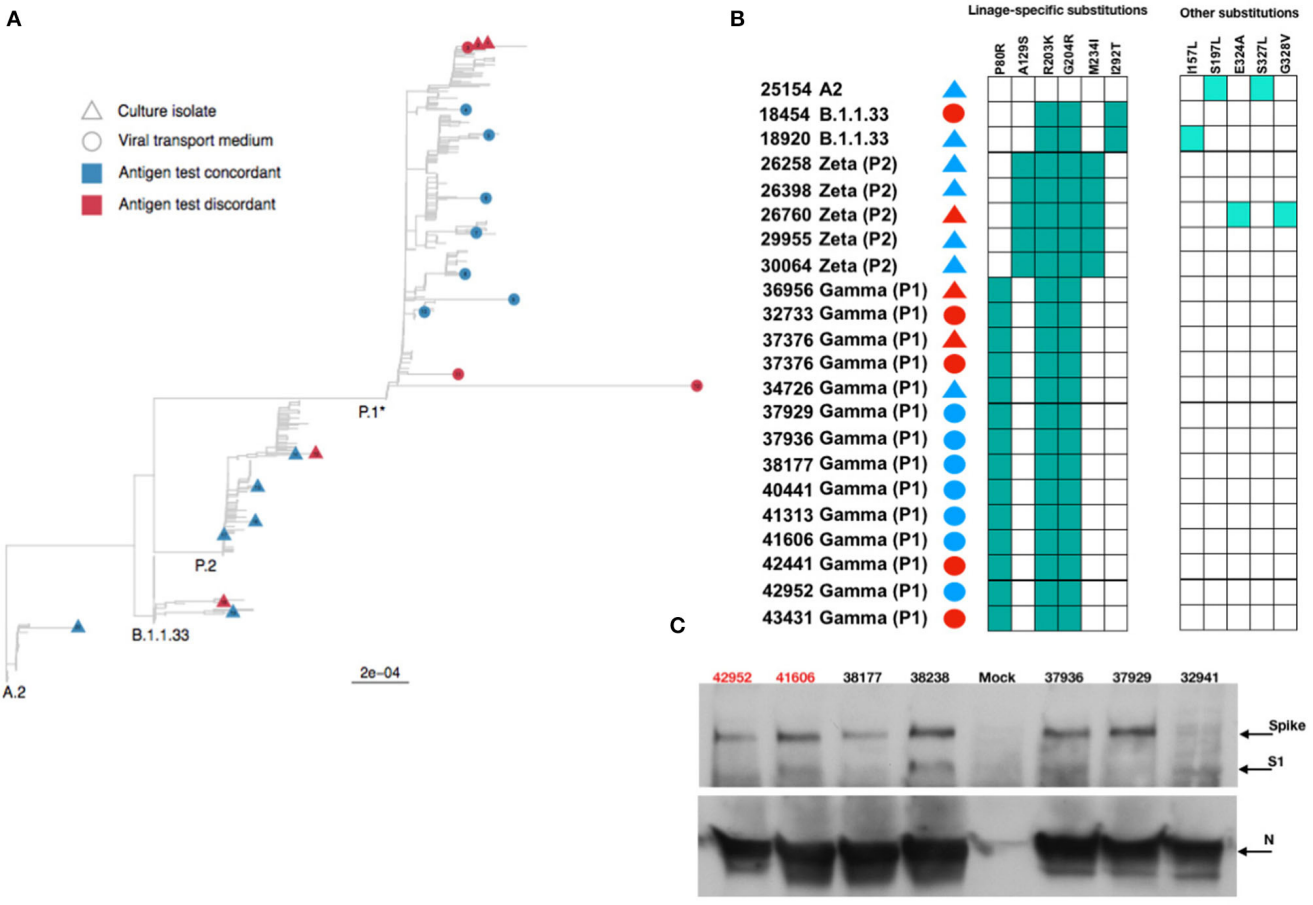
Phylogenetic and protein analysis indicate no major differences between concordant and discordant samples. **(A)** Phylogenetic tree of the viral transport medium (VTM) and isolated viral samples demonstrated that concordant and discordant samples do not form specific clusters and are grouped only according to the viral lineage. **(B)** Schematic representation of amino acid differences in N gene between sequenced VTM or viral isolated concordant and discordant samples. **(C)** Detection of SARS-CoV-2 Spike, S1, and nucleocapsid (*N*) proteins by Western blotting in lysates from Vero E6 cells infected with concordant and discordant samples. Uninfected cells were used as mock. Antigen discordant samples (42,952 and 41,606) in red and antigen concordant samples (38,177, 38,238, 37,936, 37,929, and 32,941) in black.

Then, we used the VTM and isolated viruses to characterize the full-length genomic viral RNA from a few concordant and discordant samples. We recovered intact full-length genomes from 8 discordant (4 VTM and 4 virus isolates) and 14 concordant (7 VTM and 7 virus isolates) samples. The phylogenetic reconstruction demonstrated the clustering of these samples in 3 distinct clades: B.1.1.33; Brazilian P.2 (Zeta); and P.1 (Gamma), in agreement with the period samples were collected (Figure 5A). Distinct clustering was not observed when we analyzed discordant vs. concordant samples, suggesting that no distinct genome characteristic would account for the discordant status. Analyzing specifically the N ORF, no major mutations or insertion/deletions were present in the discordant samples, excluding the possibility that gross genomic differences could compromise the recognition of the N protein by Ag-RDT (Figure 5B), and the nucleocapsid mutation profiles were largely identical for both groups (Figure 5B). Sequences recovered from both VTM and isolated virus from discordant samples 37,376 were identical.

Both S and N proteins were readily detected from infected cell lysates by Western blotting, and no major difference in S and N amounts was observed (Figure 5C). The anti-N antibody equally detected the N protein from viruses recovered from both discordant and concordant samples. These data demonstrate that although discordant samples failed to be detected by the antibodies in the Ag-RDT, it was probably due to the small amount of intact viral particles in the nasopharyngeal samples rather than a lack of reactivity of kit capture monoclonal antibody with N protein.

**Figure 6:**
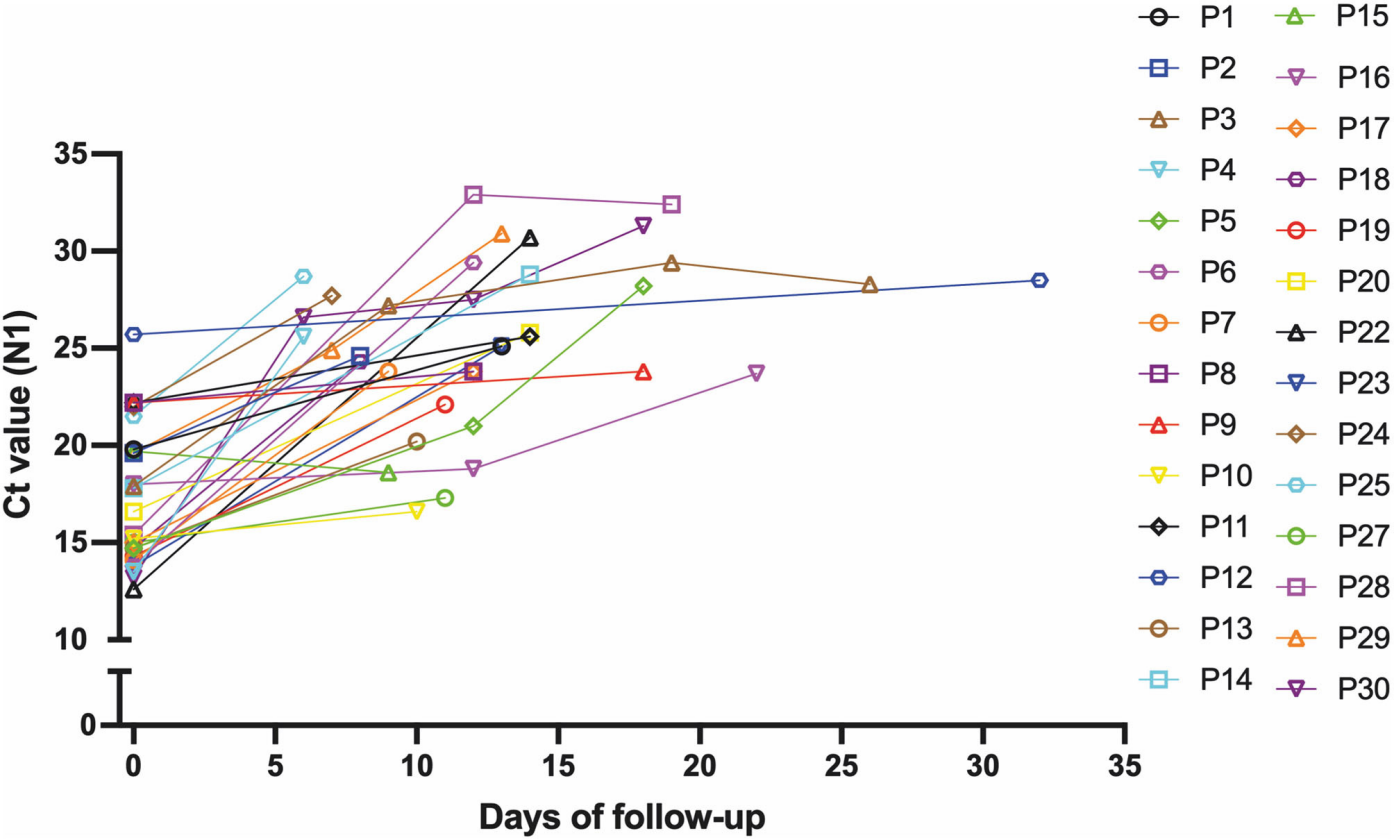
*C*_t_ values increase with prolonged infectious periods in follow-up samples. Graphical representation of the *C*_t_ value from the 30 patients that comprised the follow-up dataset at the first sample collection and the following samples from these individuals during the follow-up.

Then, we hypothesized that patients with a prolonged infection present reduced viral shedding, thus accounting for the discordance between the Ag-RDT and RT-qPCR results. To evaluate the impact of the time of infection on antigen detection, we studied a cohort of 30 patients longitudinally, totaling 41 follow-up specimens, that were collected from January to September 2021, where the first patient sample was mandatorily concordant (RT-qPCR+/Ag-RDT+) and the patient had at least one more sample collected after the first diagnosis. All sample information is summarized in Supplementary Table 6. Again, the average age and the presence of mild disease symptoms were equivalents between the concordant and discordant groups (Supplementary Table 6), and no participant had a previous diagnosis of immunodeficiency. The minimal time interval between the first (day 0) and the following sample collection varied from 6 to 32 days. On day 0, the sample *C*_t_ ranged from 12 to 26 followed by an overall increase in the *C*_t_ value for viral RNA over time (Table 1 and Figure 6). We observed that N-sgRNA values also increased, and antigen-positive results decreased as time passed since symptom onset (Table 1). Therefore, a prolonged SARS-CoV-2 infection increases the likelihood of a negative Ag-RDT result without impacting detection by RT-qPCR. Importantly, out of 5 follow-up samples that were still positive for the Antigen RDT, 3 were collected early after symptom onset (6–8 days). The VTM of 70 samples was used to infect Vero-hA/T cells, followed by plaque titration of passage #2 supernatants. Among the 29 samples from day 0, 21 produced infectious viruses (rate = 74.41%). Among the 36 discordant follow-up samples, viruses were isolated from 5 (rate = 13.89%) showing a clear drop in virus isolation with a DSSO increase (Table 1 and Supplementary Table 6). These data show that most upper respiratory tract discordant samples do not harbor infectious viruses and consequently would be less transmissible.

**Table 1:** Summarized data from follow-up samples.

	**Days after diagnosis**		
	**0**	**6–14**	**>14**
Number of samples	29	29	13
**Ct value RT-qPCR (median)**			
Genomic	17.71	27.48	35.03
Subgenomic	19.53	29.50	0.0
VTM Antigen positive result (%)	100.0	12.19	0.0
**IgG anti-S**			
Median OD	1.15	3.93	3.86
Positive results (%)	43.33 (13^*^/30)	96.29 (26/27)	83.33 (10/12)
**IgG anti-N**			
Median OD	0.70	3.70	4.30
Positive results (%)	23.33 (7/30)	92.59 (23^!^/25)	100.0 (12/12)
Virus isolation (% success)^#^	72.41 (21/29)	10.71 (3/28)	23.08 (3^&^/13)

* *Seven patients were previously fully vaccinated*.

*!The two negative samples had 6 and 7 days since symptom onset (DSSO)*.

# *Related to the total of follow-up samples (41) including concordant (5) and discordant (36) samples. The overall rate of virus isolation among the discordant samples is 13.89% (5/36)*.

& *Two isolations came from consecutive samples of a single individual*.

All viruses isolated after 2 or 3 consecutive passages were analyzed for infectious viral particles and copy numbers of genomic RNA (gRNA) in order to obtain the gRNA copy number/infectious particle relationship that would indicate the percentage of defective viruses within a viral progeny. No difference in the average of both infectious virus titers and the gRNA copy number/infectious particle relationship was observed. In fact, a wide variation in infectious virus titers was observed in both groups, and we were not able to correlate the percentage of defective progeny with viral isolates from discordant samples (Supplementary Figure 5; Supplementary Tables 7, 8).

We demonstrated that a prolonged infection period decreases the likelihood of viral particle detection by the Panbio™ Ag-RDT, which, in our data set, is overall correlated with lower viral loads in nasopharyngeal samples as well as a lower level of viral replication/infectivity. However, a few discordant samples had viral loads in the range of 18–25 which predicted a substantial amount of viral replication and the presence of infectious viruses. The establishment of the anti-SARS-CoV-2 humoral response in those individuals would contribute to a decrease in detection by the Ag-RDT. The presence of both serum IgG anti-N and anti-S antibodies was measured in initial and followed-up samples. We observed that 92.59% and 100% of samples collected 6–14 and >14 days after diagnosis, respectively, had serum anti-N IgG. Notably, 96.29 and 83.33% of samples collected 6–14 and >14 days after diagnosis, respectively, had an anti-S IgG response (Table 1). Among initial diagnostic specimens, 23.33% already had serum anti-N IgG and 43.33% anti-S IgG, while 87.88% (36/41) of the followed-up sample post-seroconverted for both serum anti-S and anti-N IgG. Individuals that had a negative Ag-RDT in 15.78% of the diagnosed patients had been previously fully vaccinated. Taken together, these data suggest that a prolonged SARS-CoV-2 infection predicts a lower viral load and virus replication in the nasopharynx associated with decreased detection by Ag-RDT.

## Discussion

Since the COVID-19 pandemic remains a public health challenge even after the rapid development and increased access to available vaccines, it is important to ensure testing capacity with fast and reliable methods to guide patient isolation to curb virus transmission. For instance, the most recent wave of the Omicron variant illustrates the need for rapid detection and isolation of infected individuals, reinforcing the need for implementing Ag-RDTs as a means of reliable, affordable, accessible, and faster SARS-CoV-2 diagnosis test.

Our study cohort was composed of two datasets: a cross-sectional dataset, where the Panbio™ COVID-19 Ag test was only performed in symptomatic patients, and a follow-up dataset in which at least two samples were collected from the same patient for the Ag-RDT with a minimum of 6 days between sample collections. For both datasets, the viral isolation rate was higher for the concordant samples (56.52–66.7%) when compared with the discordant ones (10.53–10%) even for the same *C*_t_ range (14–29 vs. 18–29). For discordant samples, viruses could not be cultured when *C*_t_ values were higher than 30, confirming previous reports of low or absence of infectious viruses at this *C*_t_ range (Bullard et al., 2020; Sonnleitner et al., 2021). We observed that the virus isolation rate is not altered by different viral variants or sample collection. In different epidemiological weeks, RT-qPCR results indicated higher *C*_t_s for discordant samples for both genomic and sgRNA compared with concordant samples, and the relationship between genomic and sgRNA demonstrated that sgRNA levels do not impact the *C*_t_ of diagnosis or the isolation success in both groups.

The virus isolation criteria used in this article rely on a plaque titration assay for the determination of positive or negative sample isolation. Thus, only samples harboring replication-competent viruses were considered, which is a proxy for specimen infectivity, providing a more reliable measurement of viral viability/infectiousness than RT-qPCR. Our results demonstrated that discordant samples present lower levels of viral replication meaning that a patient with a negative Ag-RDT result has a reduced risk of transmission even when presenting as RT-qPCR positive, indicating that Ag-RDT has high epidemiological relevance.

Then, the use of Ag-RDTs could be a powerful tool for detecting infected individuals with a higher potential for transmission. Its specificity and sensitivity correlate with time from symptom onset and the presence of viral infectious particles. Data on the use of antigen tests in large cohorts such as universities, healthcare institutions, or even in music events as a SARS-CoV-2 screening method demonstrated that sensitivity depends on the target population where the test is used. In general population screening, antigen tests had a reported sensitivity from 41.2 to 74.4% in different countries (Landaas et al., 2021; Loconsole et al., 2021; Shaw et al., 2021), and in emergency rooms and nursing homes, the sensitivity varied from 50 to 92% even for asymptomatic individuals (Escrivá et al., 2021; Turcato et al., 2021). Revollo et al. demonstrated the utility of Ag-RDTs for the same day screening in mass gathering events, where no infected individuals were detected in follow-up tests 8 days after the event, indicating that the antigen test can detect potential transmitters (Revollo et al., 2021). Despite the variation in sensitivity, a recent study using mathematical modeling showed that Ag-RDTs could be more efficient than RT-qPCR in reducing SARS-CoV-2 transmission by up to 85% in a nursing home facility (Holmdahl et al., 2021). Another study predicts that Ag-RDTs could prevent infections better than RT-qPCR in a workplace (Pettit et al., 2021).

As vaccination campaigns progress around the world, high vaccine coverage in several regions has been achieved (World Health Organization, 2020). Nevertheless, places such as continental Europe and the US are experiencing new waves of infection with thousands of new cases daily. Although vaccines present a >90% protection against severe disease (Fabiani et al., 2021; Glatman-Freedman et al., 2021), their effectiveness has decreased substantially (Polack et al., 2020; Bar-On et al., 2021). One of the factors related to the decrease in effectiveness is the emergence of new SARS-CoV-2 variants. An effectiveness reduction from 91.78 to 79.87% was reported in New York City from May to July 2021 as well as the detection of breakthrough cases among fully vaccinated individuals infected with the Delta variant (Brown et al., 2021; Rosenberg et al., 2021).

New variants are mainly classified according to mutations in Spike protein; however, since mutations also accumulate in the N gene, they could impact the results of Ag-RDTs targeting N. The samples used in this study were collected throughout waves of different variants that circulated in Rio de Janeiro from August 2020 to September 2021, including the Zeta, Gamma, and Delta lineages. Our results agree with others that did not observe any impact on Ag detection of these variants (Bekliz et al., 2021; Rodgers et al., 2021). The presence of substitutions T205I and D399N in the N ORF was linked to a failure in Ag-RDT detection (Bourassa et al., 2021). We observed few mutations in concordant and discordant samples, but none impacted antigen recognition. The N and S proteins from viruses isolated from discordant samples were readily detected by Western blot, emphasizing the fact that when present in adequate amounts and/or conditions, and no difference in antigen-antibody recognition for all samples will exist.

It was clear that a specific and still unrecognized condition of the discordant sample would account for the Ag-RDT negative result and lower probability of virus isolation even with the presence of high amounts of intact full-length viral RNA. The characterization of samples in the follow-up dataset narrowed down this specific condition to a phenomenon related to time past from initial symptoms, which could be for instance patient's seroconversion, although the presence of circulating serum anti-N and anti-S IgG, measured in this study, does not necessarily correlate with mucosal immunoglobulins. Thus, the establishment of an upper respiratory tract environment over the time of infection could induce virus aggregation/reduced stability leading to low viral shedding and missed antigen capture in Ag-RDT.

Recent data demonstrated a higher rate of disease progression in individuals with no detectable antibody and, as our results indicate, the disappearance of antigen and infectious virus from the upper respiratory tract correlated with the rise of anti-SARS-CoV-2 antibodies (Vetter et al., 2020; Glans et al., 2021; Kim et al., 2021; Lv et al., 2021; van Kampen et al., 2021). These data suggest that a positive Ag-RDT indicates active disease and a longer time for viral RNA clearance than antigen. Considering the variable Ag-RDT sensitivity, its implementation for mass rapid testing is valuable to identify highly infectious individuals in a community, as most Ag-RDT negative samples probably will not harbor infectious viruses. Therefore, it may assist health authorities to control the virus spread while avoiding unnecessary individual isolation precautions.

Previous studies analyzed a variable number of RT-qPCR + /Ag-RDT+ concordant and RT-qPCR + /Ag-RDT- discordant samples for virus isolation and demonstrated a lower probability for the presence of cultivable viruses in the later (Albert et al., 2021; Prince-Guerra et al., 2021; Toptan et al., 2021; Yamamoto et al., 2021; Currie et al., 2022). We cultivated only 24% of the qRT-PCR(+)/Ag-RDT(–) discordant samples in our cohort in the defined period. However, only one study analyzed a similar number of discordant samples and found a similar virus isolation rate (Currie et al., 2022). Another study analyzed a higher number of discordant samples, 124 qRT-PCR(+)/Ag-RDT(–) samples, and 147 truly concordant samples, obtaining an 8.9 vs. 57.8% of infectious virus isolation rate (Prince-Guerra et al., 2021). In this study, we analyzed a total of 70 qRT-PCR(+)/Ag-RDT(–) discordant and 52 truly concordant samples with infectious virus isolation rates of 12.95 vs. 63.5%, confirming the lower probability of qRT-PCR(+)/Ag-RDT(–) discordant samples to harbor infectious viruses. However, the unique way our study was designed allowed us to establish that the main factors contributing to qRT-PCR(+)/Ag-RDT(–) discordant results are RT-qPCR *C*_t_ value and the time from initial infection, which in turn reinforces the low probability of virus transmission from these samples.

## Conclusion

This study found a clear association between a negative Ag-RDT result and the absence of infectious virus in COVID-19 nasopharyngeal exudates. A positive Ag-RDT result was also correlated with a *C*_t_ <30 in our RT-qPCR test, accompanied by the absence of measurable IgG against the S and N viral proteins and <7 days after symptom onset. Our data also support the proposal to identify patients using Ag-RDT testing in the general population to substantially reduce the number of infected individuals with a higher risk of viral transmission while avoiding unnecessary individual isolation. Massive scaling of testing with Ag-RDT and patient isolation together with an extensive vaccination campaign should reduce the prevalence of SARS-CoV-2 infection and, consequently, the number of severe cases needing hospitalization, leading to a lower COVID-19 death toll.

## Data Availability Statement

The sequence data presented in the study are deposited in the GISAID repository (https://www.gisaid.org), accession numbers EPI_ISL_11836071 through yEPI_ISL_11836089 and EPI_ISL_4413334 - EPI_ISL_4413337. All other datasets are provided in the Supplementary Tables.

## Ethics Statement

The studies involving human participants were reviewed and approved by Brazilian National Committee of Research Ethics (CAAE-30161620.0.1001.5257). The patients/participants provided their written informed consent to participate in this study.

## Author Contributions

IC: research design, carried out experiments, analyzed and discussed results, and wrote the manuscript. DF and RG: research design, analyzed and discussed results, and implemented the logistic regression models. CG, RM, and GS: carried out experiments and analyzed results. FM conduct sequencing and sequence analyses. DM, MFC, and IL: sample collection, handling and storage coordination, and data collection. MS and MSC: carried out experiments and analyzed and discussed results. ÉN, LR, TFCC, and CP: sample collection, handling and storage, and carried out experiments. LG, GC, and JH: research conception, data analyses and discussion, and manuscript writing. MR and MB: carried out experiments, data analyses and discussion, and manuscript writing. RV: data analyses and discussion and manuscript writing. OF: research design, data analyses, and manuscript supervision. TMPPC: research design, data collection and analyses, and manuscript supervision. AT and LC: research conception and design, study implementation, data analyses and discussion, and manuscript supervision and editing. All authors contributed to the article and approved the submitted version.

## Funding

This work was supported by Conselho Nacional de Desenvolvimento Científico e Tecnológico; MCTI-Rede Corona-Omica-BR; Fundação Carlos Chagas Filho de Amparo à Pesquisa do Estado do Rio de Janeiro, Coordenação de Aperfeiçoamento de Pessoal de Nível Superior, Instituto Serrapilheira, Ministério da Ciência, Tecnologia e Inovações, Coordenação de Projetos, Pesquisas e Estudos Tecnológicos, and Universidade Federal do Rio de Janeiro. Abbott - Chicago–USA.

## Conflict of Interest

LG, MR, MB, RV, GC, and JH were employed by Abbott Laboratories Inc. The remaining authors declare that the research was conducted in the absence of any commercial or financial relationships that could be construed as a potential conflict of interest.

## Publisher's Note

All claims expressed in this article are solely those of the authors and do not necessarily represent those of their affiliated organizations, or those of the publisher, the editors and the reviewers. Any product that may be evaluated in this article, or claim that may be made by its manufacturer, is not guaranteed or endorsed by the publisher.

## Supplementary Material

Supplementary Table 1 GISAID accession codes and metadata information for sequences generated in this work.

Supplementary Table 2 Descriptive demographic variables by concordant (RT-qPCR+/Ag-RDT+) and discordant (RT-qPCR+/Ag-RDT-) groups.

Supplementary Table 3 Multivariate logistic regression model for demographic variables in concordant (RT-qPCR+/Ag-RDT+) and discordant (RT-qPCR+/Ag-RDT-) groups.

Supplementary Table 4 The individual demographic for total number of SARS-CoV-2 diagnosed patients from August 2020 to September 2021.

Supplementary Table 5 RT-qPCR, Ag-RDT, virus isolation and demographic information for concordant and discordant samples further analyzed.

Supplementary Table 6 RT-qPCR, Ag-RDT, virus isolation and demographic information for the follow-up group.

Supplementary Table 7 Virus titers and genomic RNA (gRNA) copy numbers.

Supplementary Table 8 Virus titers and genomic RNA (gRNA) copy numbers from follow-up samples.
