# Supplementary material for: A SARS-CoV-2 Negative Antigen Rapid Diagnostic in RT-qPCR Positive Samples Correlates With a Low Likelihood of Infectious Viruses in the Nasopharynx
Source: Front Microbiol. 2022 Jul 27;13:912138. doi: 10.3389/fmicb.2022.912138 (PMC9364907; doi:10.3389/fmicb.2022.912138)
Supplement: Supplementary Table 2 — Descriptive demographic variables by concordant (RT-qPCR+/Ag-RDT+) and discordant (RT-qPCR+/Ag-RDT-) groups. [file Table_2.DOCX]

Supplementary Table 2: Descriptive demographic variables by concordant (RT-qPCR+/Ag-RDT+) and discordant (RT-qPCR+/Ag-RDT-) groups.

0 = discordant Ag-RDT-/RT-qPCR+; 1 = concordant Ag-RDT+/RT-qPCR+
