# Supplementary material for: A SARS-CoV-2 Negative Antigen Rapid Diagnostic in RT-qPCR Positive Samples Correlates With a Low Likelihood of Infectious Viruses in the Nasopharynx
Source: Front Microbiol. 2022 Jul 27;13:912138. doi: 10.3389/fmicb.2022.912138 (PMC9364907; doi:10.3389/fmicb.2022.912138)
Supplement: Supplementary Table 7 — Virus titers and genomic RNA (gRNA) copy numbers. [file Table_7.DOCX]

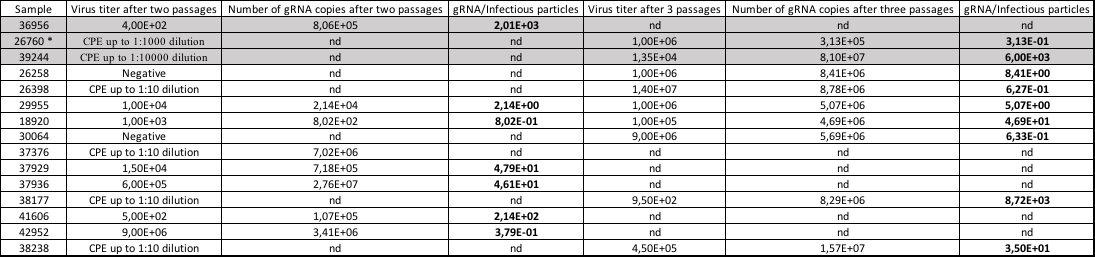
Supplementary Table 7: Virus titers and genomic RNA (gRNA) copy numbers

* Copy number calculated from the N gene was 26-times lower than that obtained for the Orf gene (8.29 x 10^6^). Using the copy number from the Orf gene the gRNA/infectious particle relationship is 8.29.

Discordant samples are highlighted.


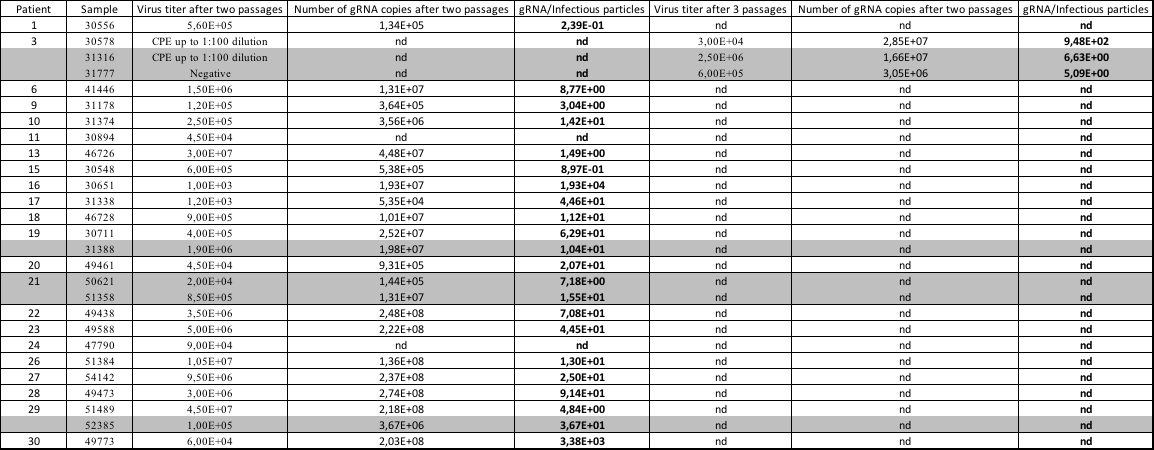
Supplementary Table 8: Virus titers and genomic RNA (gRNA) copy numbers from follow-up samples

Discordant samples are highlighted.
