# Supplementary material for: A SARS-CoV-2 Negative Antigen Rapid Diagnostic in RT-qPCR Positive Samples Correlates With a Low Likelihood of Infectious Viruses in the Nasopharynx
Source: Front Microbiol. 2022 Jul 27;13:912138. doi: 10.3389/fmicb.2022.912138 (PMC9364907; doi:10.3389/fmicb.2022.912138)
Supplement: Supplementary file 8 [file Data_Sheet_1.docx]

**Supplementary Figures:**


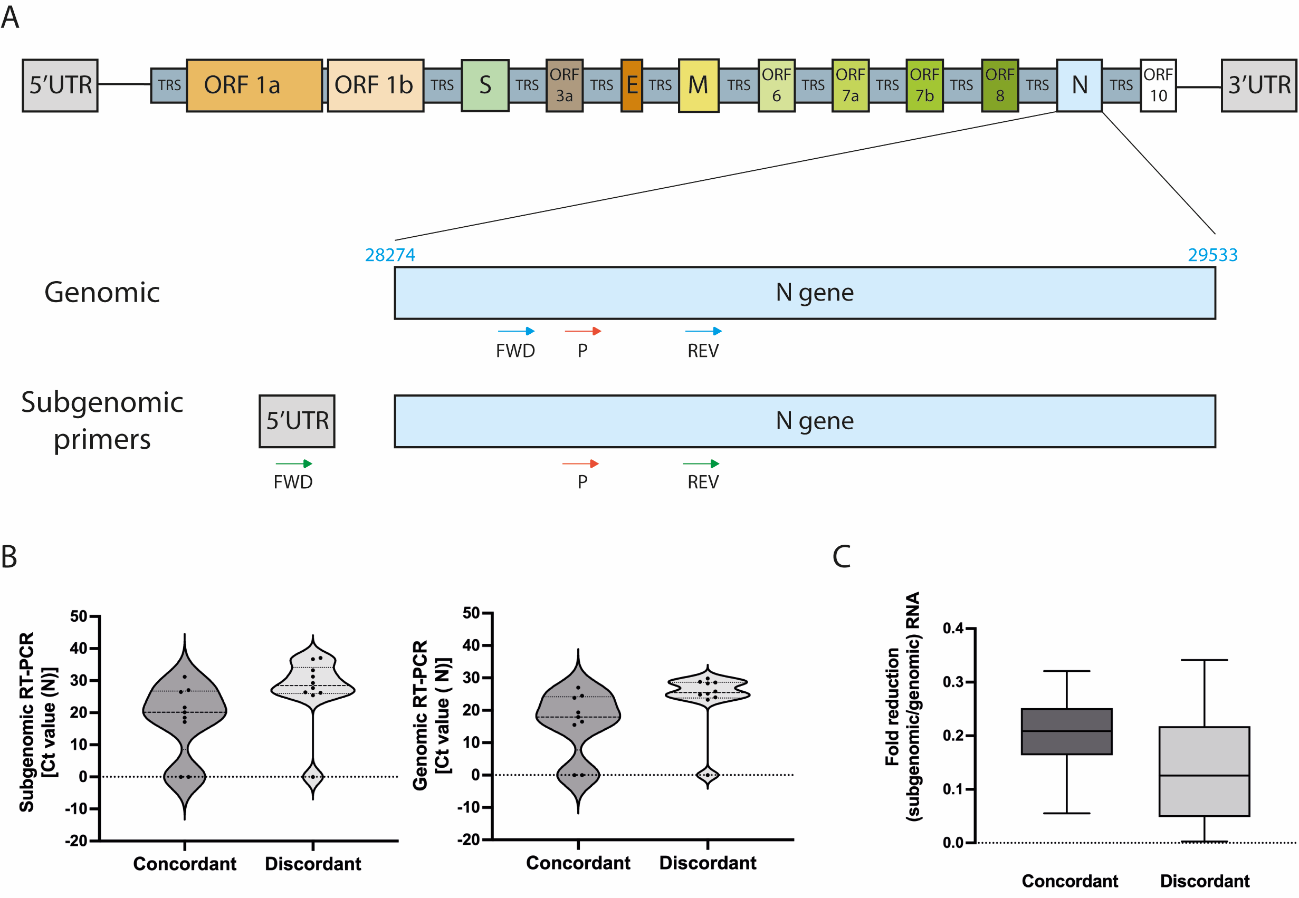


**Supplementary Figure 1. Antigen concordant and discordant samples had similar genomic to subgenomic RNA ratio.** *A:* Schematic representation of primers and probes used for RT-qPCR detection of genomic and subgenomic RNA in VTM samples. Primer sequences used for genomic detection are N_sarbeco_F - CACATTGGCACCCGCAATC and N_sarbeco_R - GAGGAACGAGAAGAGGCTTG. For subgenomic detection, primers sequences are: FWSGRNAN - CGATCTCTTGATCTGTTCTCTAAACGAACAAATTAAAATG and N_sarbeco_R - GAGGAACGAGAAGAGGCTTG. For both reactions, the probe used was N_sarbeco_P - FAM-ACTTCCTCAAGGAACAACATTGCCA-BBQ. *B:* Median Ct value from genomic RNA (N gene) from antigen positive and negative VTM samples. *C:* Fold reduction calculated based on the delta Ct of genomic and subgenomic ratio.

**
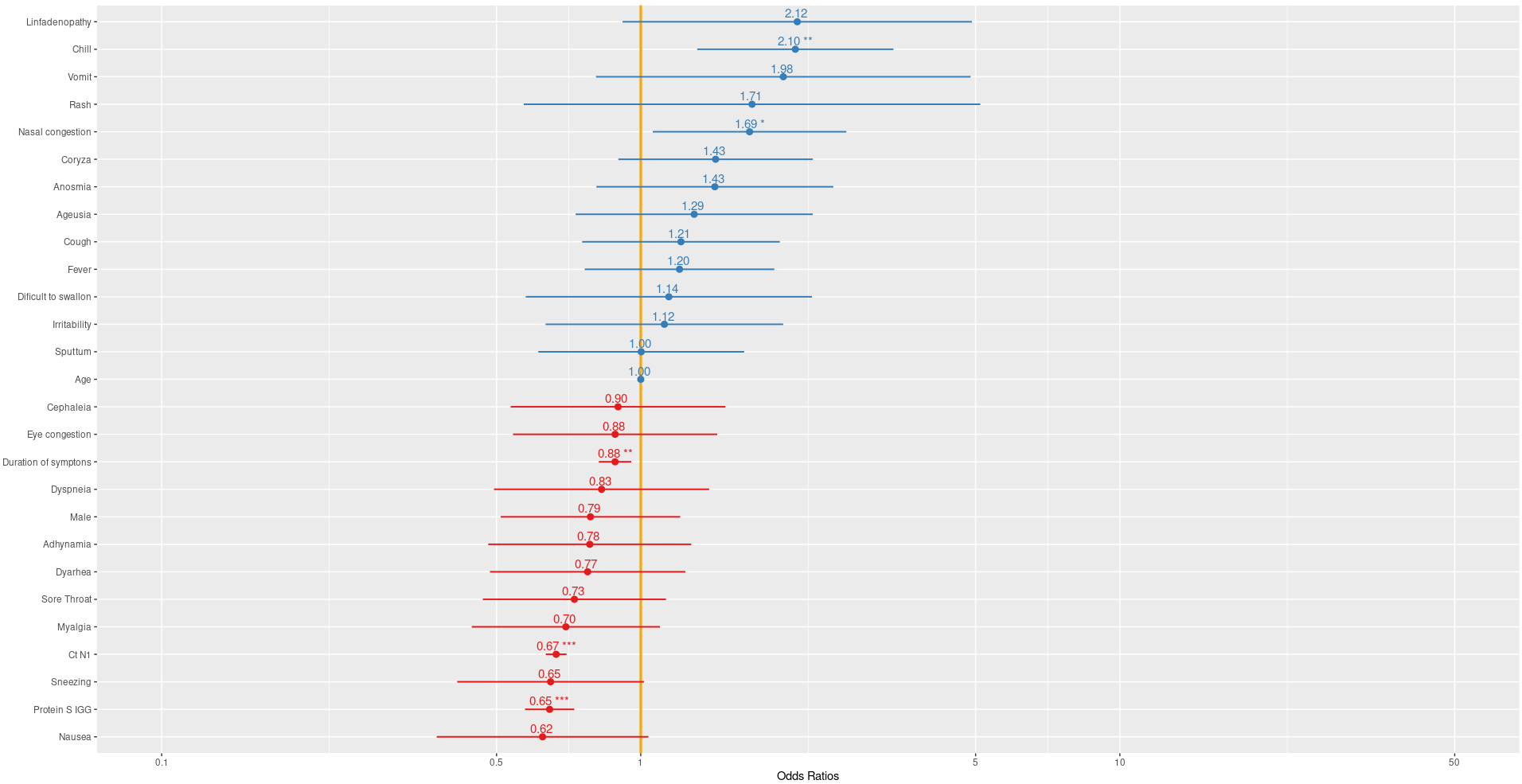
**

**Supplementary Figure 2.** Forest plot showing the odds ratios of factors associated with discrepant results (Ag-RDT-/RT-qPCR+) for SARS-CoV-2 between Ag-RDT and RT-qPCR tests. Horizontal lines represent 95% Confidence Interval.


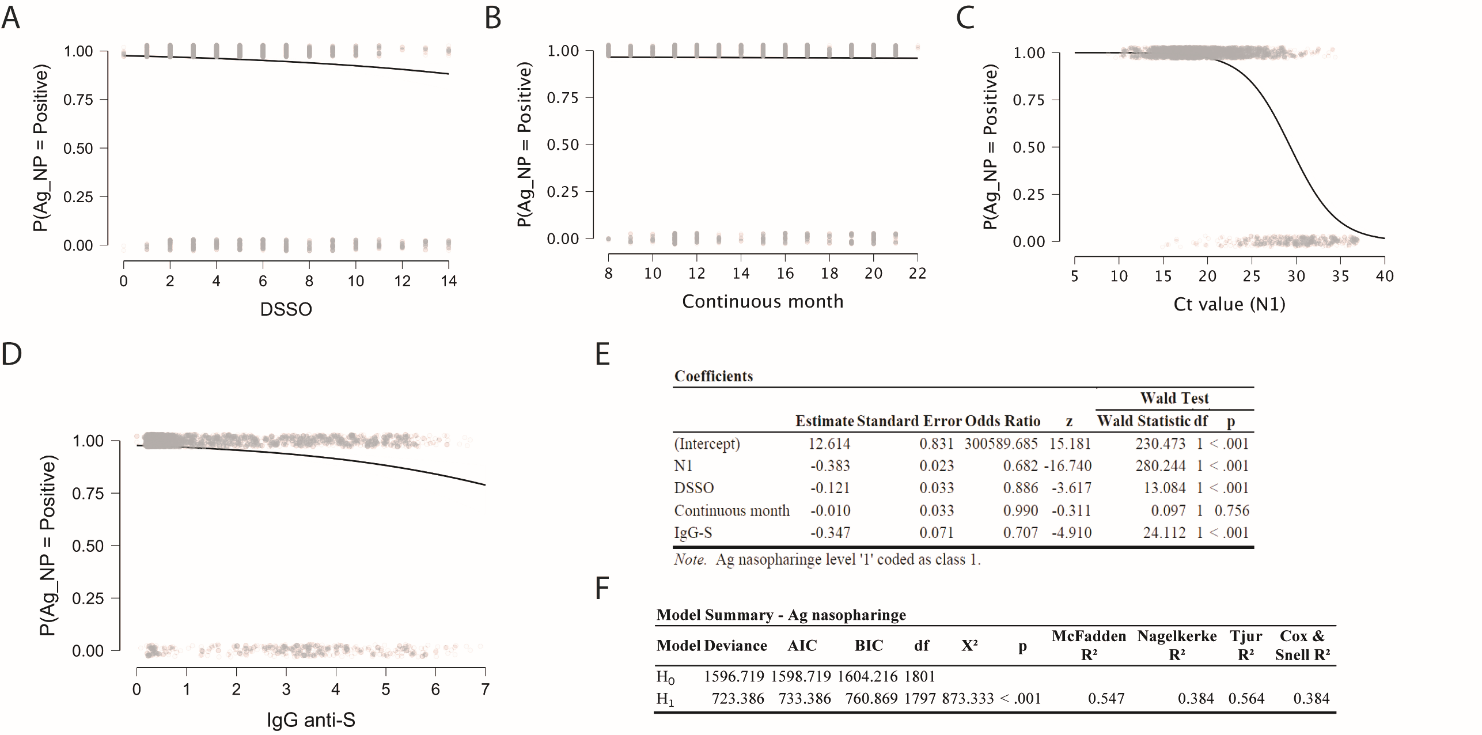


**Supplementary Figure 3. SARS-CoV-2 RT-PCR Ct value and IgG anti-S levels highly influence the chance of having a positive nasopharyngeal antigen test result.** Logistic regression showing the probability of having a positive nasopharyngeal antigen (Ag_NP) result according to: (A) SARS-CoV-2 RT-PCR Ct value (N1 target); (B) IgG anti-S level; (C) days since symptom onset; and (D) month of sample collection, from Ago 2020 to Sep 2021 (continuous month 8-21). (E and F) model summary and coefficients of logistic regression showing the probability of having a positive nasopharyngeal antigen (Ag_NP) result according to SARS-CoV-2 RT-PCR Ct value (N1 target); IgG anti-S level; days since symptom onset; and month of sample collection, from Aug 2020 to Sep 2021 (continuous month 8-21).


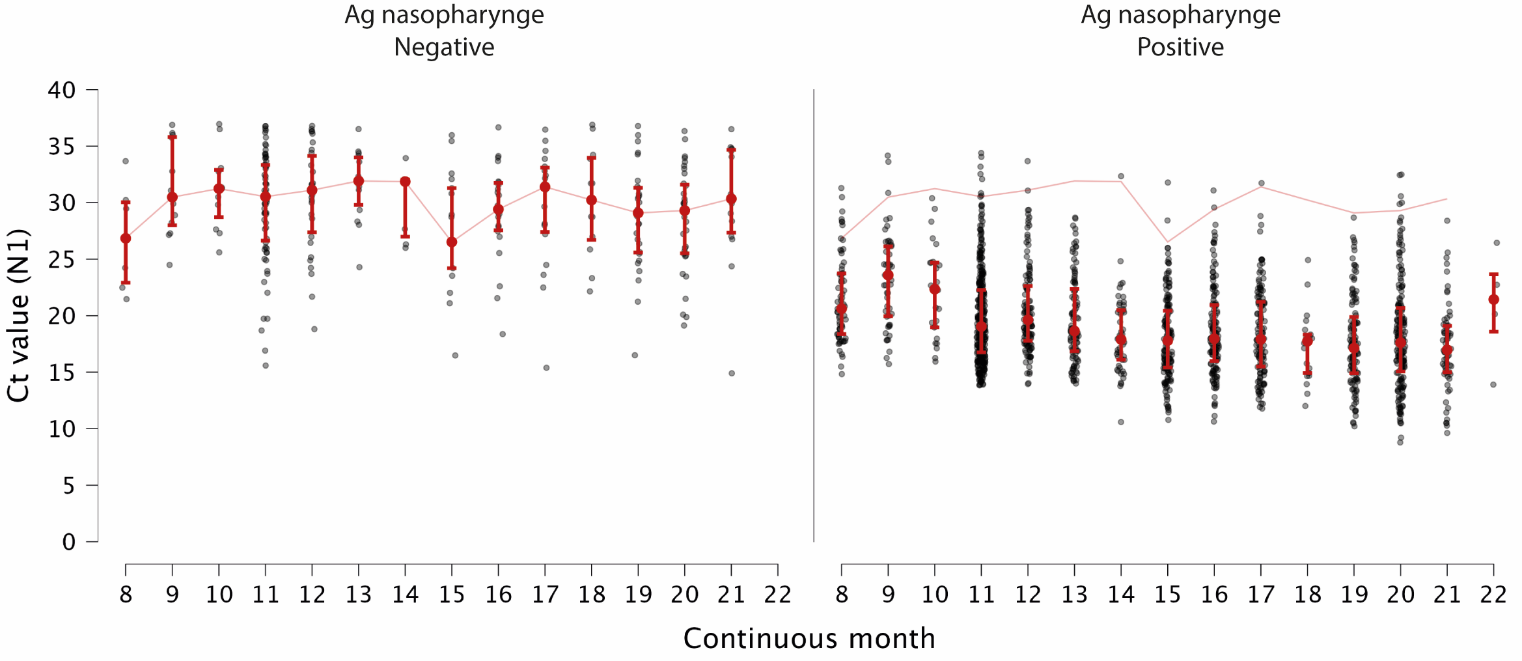


**Supplementary Figure 4.** **Continuous analysis of C_t_ values from samples RT-qPCR positive results demonstrated that samples with antigen RDT positive results had lower Ct values than antigen RDT negative ones.** Ct values (N1 target) of SARS-CoV-2 RT-qPCR in patients with positive or negative Ag-RDT results by month, from August 2020 to September 2021 (continuous month 8-21).


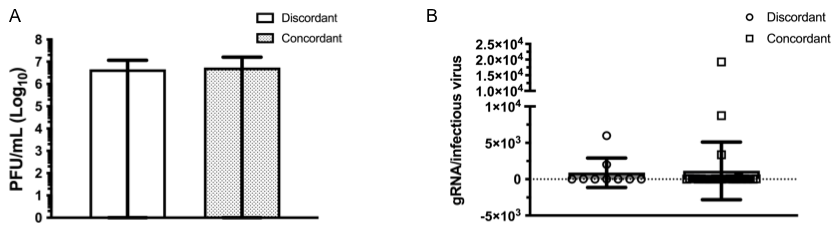


**Supplementary Figure 5. Virus titers and viable gRNA measurements for viruses isolated from concordant and discordant samples.** *A:* Infectious virus titers from isolated viruses in second or third passages from both discordant and concordant samples showing a similar average titer for both groups, but a great variability in the amount of infectious virus from viral isolates from both groups. *B:* The percentage of non-infectious gRNA (or defective viral particles) in a viral stock can be obtained from the gRNA copy number/infectious virus titers (in PFU/mL). The average of defective viral particles percentage analyzing isolated viruses obtained from discordant and concordant samples is similar, while the greatest and the lowest number of defective particles (> 10^4^: 1 viral isolate and 1: 5 viral isolates) were observed in viruses obtained from concordant sample. Thus, 17.8% (5/28) viral isolates from concordant samples are highly efficient in producing fully infectious viruses.
